# High‐density molecular characterization and association mapping in Ethiopian durum wheat landraces reveals high diversity and potential for wheat breeding

**DOI:** 10.1111/pbi.12538

**Published:** 2016-02-08

**Authors:** Dejene Kassahun Mengistu, Yosef Gebrehawaryat Kidane, Marcello Catellani, Elisabetta Frascaroli, Carlo Fadda, Mario Enrico Pè, Matteo Dell'Acqua

**Affiliations:** ^1^ Institute of Life SciencesScuola Superiore Sant'AnnaPisaItaly; ^2^ Department of Dryland Crop and Horticultural SciencesMekelle UniversityMekelleEthiopia; ^3^ Sirinka Agricultural Research CenterSirinka, WoldiaEthiopia; ^4^ Department of Agricultural SciencesUniversity of BolognaBolognaItaly; ^5^ Bioversity InternationalC/O International Livestock Research Institute (ILRI)Addis AbabaEthiopia; ^6^ Present address: ENEA, UT‐BIORADLaboratory of BiotechnologyResearch Center CasacciaVia Anguillarese 30100123RomeItaly

**Keywords:** *Triticum turgidum* subsp. *durum*, GWAS, population genetics, quantitative trait loci, Ethiopia, linkage disequilibrium

## Abstract

Durum wheat (*Triticum turgidum* subsp. *durum*) is a key crop worldwide, and yet, its improvement and adaptation to emerging environmental threats is made difficult by the limited amount of allelic variation included in its elite pool. New allelic diversity may provide novel loci to international crop breeding through quantitative trait loci (QTL) mapping in unexplored material. Here, we report the extensive molecular and phenotypic characterization of hundreds of Ethiopian durum wheat landraces and several Ethiopian improved lines. We test 81 587 markers scoring 30 155 single nucleotide polymorphisms and use them to survey the diversity, structure, and genome‐specific variation in the panel. We show the uniqueness of Ethiopian germplasm using a siding collection of Mediterranean durum wheat accessions. We phenotype the Ethiopian panel for ten agronomic traits in two highly diversified Ethiopian environments for two consecutive years and use this information to conduct a genome‐wide association study. We identify several loci underpinning agronomic traits of interest, both confirming loci already reported and describing new promising genomic regions. These loci may be efficiently targeted with molecular markers already available to conduct marker‐assisted selection in Ethiopian and international wheat. We show that Ethiopian durum wheat represents an important and mostly unexplored source of durum wheat diversity. The panel analysed in this study allows the accumulation of QTL mapping experiments, providing the initial step for a quantitative, methodical exploitation of untapped diversity in producing a better wheat.

## Introduction

Wheat is the second largest cereal commodity grown worldwide, with ever‐increasing land allocation and production (http://faostat3.fao.org). Although most wheat production is bread wheat (*Triticum aestivum* L.), the allotetraploid macaroni or durum wheat (*T. turgidum* subsp. *durum*) is a key resource for both sustenance and high‐value food production. *Triticum turgidum* subsp. *durum* breeding was pioneered in Italy, the USA and eastern Europe, when different, early landraces were introduced into breeding programmes (Royo *et al*., 2009). The pace of durum improvement increased during the 1970s, when CIMMYT started releasing internationally tested varieties (Maccaferri *et al*., 2008; Sauer, 1993). Interest in durum wheat breeding is burgeoning and, similarly to bread wheat, has been supported by the increased availability of genomic tools (Tuberosa and Pozniak, 2014). After the release of good‐quality genome drafts (Brenchley *et al*., 2012; Mayer *et al*., 2014), a high‐quality reference sequence for bread wheat, also useful for durum wheat, is under production (http://www.wheatgenome.org).

Still, an inherently complex polyploid genome such as wheat's poses serious challenges in developing single nucleotide polymorphisms (SNPs) markers, the base of tailored durum wheat improvement. Genotyping through next‐generation sequencing methods may be considered even in large genomes, if using restriction‐enzyme based approaches such as RAD sequencing and GBS (Davey *et al*., 2011; Rowe *et al*., 2011). However, polyploidy poses the risk of miscalling diversity on homoeologous genomes (Paux *et al*., 2012; Saintenac *et al*., 2013). An alternative approach is to exploit hybridization arrays, whose power recently escalated 10‐fold in wheat moving from 9k (Cavanagh *et al*., 2013) to 90k genomewide SNPs (Wang *et al*., 2014b). Tailored clustering algorithms are used on arrays to reduce the interference of paralogous loci and duplicated SNPs in correct allele calling (Akhunov *et al*., 2009; Wang *et al*., 2014b).

Dense molecular data allow quantitative genetics to more efficiently describe quantitative trait loci (QTL) at the base of wheat traits and empower marker‐assisted selection (MAS) increasing the intergeneration genetic gain (Xu *et al*., 2012). QTL mapping is historically performed on structured panels, that is recombinant lines originating by the intercrosses of two or more inbred lines (Cui *et al*., 2013; Huang *et al*., 2012) also allowing the creation of a genetic reference map (Maccaferri *et al*., 2015). However, recent advances in statistical methods named genome‐wide association (GWA) studies allow to consider diversity panels of unrelated individuals as QTL mapping resources (Yu *et al*., 2006; Zhang *et al*., 2010; Zhou and Stephens, 2012). GWA approaches provide significant data for QTL mapping and MAS applications, through the low‐cost, high‐throughput identification of useful alleles in any collection of germplasm (Lopes *et al*., 2014; Maccaferri *et al*., 2011; Sukumaran *et al*., 2015).

Allelic and phenotypic variations are the raw material on which GWA statistical associations are drawn: when dealing with crops, both may not be a given. Wheat underwent several diversity bottlenecks, from domestication until modern agronomic improvements (Haudry *et al*., 2007; Peng *et al*., 2011). The consequent erosion of genetic diversity limits the pool of alleles in which to search for new traits of agronomic interest, constraining elite lines improvement perspectives and capacity to deal with threats such as climate change and land limitation (Grassini *et al*., 2013). Landraces might be a ready response to revert this trend by introducing novel variation in established, elite backgrounds (Feuillet *et al*., 2008; Lopes *et al*., 2015). Wheat landraces may contribute traits including pest and pathogen resistance (Cavanagh *et al*., 2013; Ghavami *et al*., 2011), abiotic stress resistance (Lopes *et al*., 2015; Olmstead and Rhode, 2002), and gluten quality and composition (Moragues *et al*., 2005; Zheng *et al*., 2012).

Durum wheat landraces have been actively maintained in several traditional agro‐ecosystems of the Old World, including Morocco (Sahri *et al*., 2014), Turkey (Akar and Ozgen, 2007), Jordan (Rawashdeh *et al*., 2007) and Iran (Mohammadi *et al*., 2014). However, none of these regions probably matches the landrace reservoir in Ethiopia, a centre of extraordinary wheat diversity (Harlan, 1969; Vavilov, 1992). Ethiopian durum wheat early attracted the wheat community because of its uniqueness under several morphological aspects (Sakamoto and Fukui, 1972; Porceddu *et al*. 1973; Pecetti *et al*., 1992); the number of landraces that Ethiopia harnesses is matched by the different environmental conditions in which they are grown (Pecetti and Damania, 1996; Tesemma and Belay, 1991). Ethiopian durum wheat landraces are cultivated in a small‐to‐medium scale traditional farming system, the most diffused in the country. Families and villages informally exchange germplasm that shows huge genetic variations in several valuable traits (Gashaw *et al*., 2007; Hailu *et al*., 2010; Teklu and Hammer, 2009). The genetic variability in Ethiopian durum wheat has already been characterized by microsatellite markers, confirming an elevated amount of variation (Alamerew *et al*., 2004; Haile *et al*., 2013; Teklu *et al*., 2006). To date, these efforts have only scratched the surface of the breeding resources represented by Ethiopian durum wheat landraces.

The aim of this study was to provide the most thorough characterization of Ethiopian durum wheat landraces performed to date. We conducted SNP genotyping on more than 80 000 loci in a broad collection of Ethiopian durum wheat to provide a description of Ethiopian material diversity in terms of improved wheat as well as between internally displaced landraces. To enable a GWA approach, the genotyped accessions were phenotyped for two subsequent years (2012–2013) at two locations in Ethiopia, collecting data for three phenological and seven agronomic traits. We performed a GWA scan to report chromosomal regions that influence the agronomic performance of Ethiopian durum wheat under local growing conditions. By this, we aim to provide the first step in a full evaluation of the potential of Ethiopian durum wheat landraces in international breeding.

## Results

### Genotyping and species assignation

**Figure 1:**
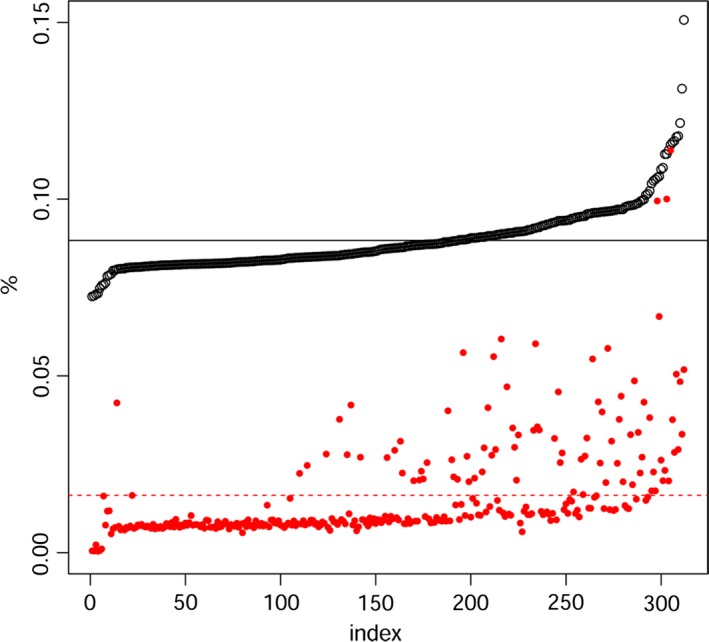
Failure rate (black dots) and heterozygosity (red dots) for Ethiopian durum wheat samples. Samples are ordered by increasing failure rate. Mean values of the two distributions are reported as broken lines. Note that as failure rate increases, so does heterozygosity, possibly because of less than optimal annealing of the array probes.

Our collection of Ethiopian wheat is composed of 311 Ethiopian durum wheat accessions, of which 24 are improved varieties approved for cultivation in Ethiopia. The Ethiopian data set was sided by 38 durum wheat accessions and lines cultivated around Mediterranean basin (Table S1). Of the 81 587 probes available on the chip, 2899 consistently failed, that is did not produce information about the allele at that specific locus. Average heterozygosity in the Ethiopian durum wheat data set was equal to 0.02, and failure rate (‘N’ calls over the total) averaged 0.15 (Figure 1). As failure rates increased, so did heterozygosity. The two are possibly related by problematic probe hybridizations. Both heterozygosity and failure rates were consistently higher in Ethiopian than in Mediterranean durum wheat (Figure S1), especially in the top outliers. Probes failing in more than 25% of the samples were removed as possible indicators of markers with a low specificity. The array scored 30 155 polymorphic loci in the Ethiopian durum wheat subset. The same array scored 21 069 polymorphic SNPs when considering Mediterranean material alone.

### Molecular diversity analyses

**Figure 2:**
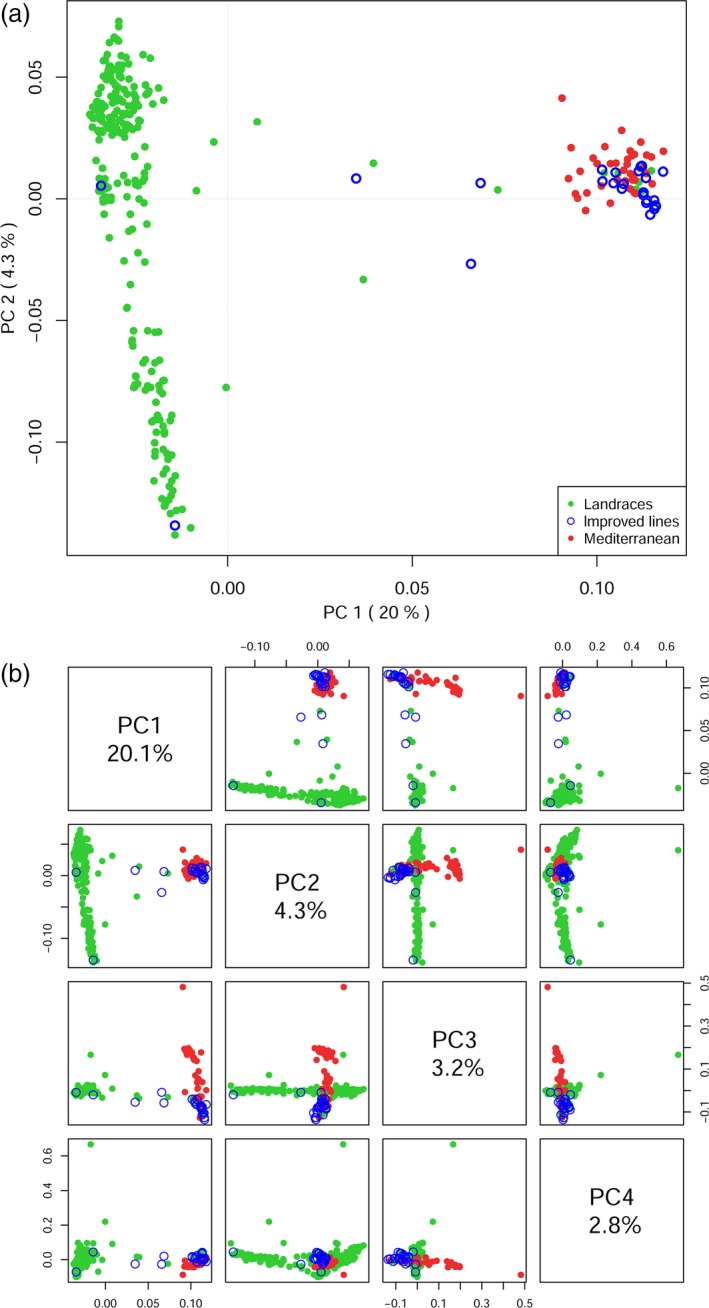
Principal component analysis of genetic diversity within the data set. (a) Ethiopian durum wheat landraces (green) are clearly separated from Mediterranean durum wheat (red) by the PC axis with the highest significance (20%). Improved varieties registered for cultivation in Ethiopia (blue) cluster with the Mediterranean material with few exceptions. (b) PC 1–4, colour code is the same as in panel a. Note the low variance explained by the PC axes.

We performed a principal component analysis (PCA) on the SNPs data of Ethiopian and Mediterranean durum wheat samples (Figure 2a). The first and second PCs accounted for 20.1% and 4.3% of the variance, respectively. Many of the improved varieties approved for cultivation in Ethiopia clustered together with Mediterranean accessions and were separated from Ethiopian landraces by PC1. Three samples classified as improved varieties clustered with landraces, and an additional three laid intermediate between the two clusters. Seven landraces grouped with the Mediterranean samples. This arrangement was disrupted when additional principal components were considered (Figure 2b). The already low variance explained by the second PC shows that Ethiopian landrace diversity cannot be summarized by one or a few PCs. A neighbour‐joining (NJ) phylogeny confirmed that Ethiopian improved varieties are very similar and clearly distinct from the bulk of landraces. The long edges of the phylogeny confirm a high, poorly structured diversity between landraces (Figure S2). We did not find a relation between the year of release of the improved varieties and their placement in the phylogeny.

Loci polymorphic in the Ethiopian data set were inputted into a Bayesian structure analysis to estimate the number of cryptic genetic clusters in the panel. The great diversity between improved varieties and landraces, which emerged in the previous analyses, yielded a strong signal for the existence of two distinct genetic clusters (data not shown). When improved varieties were removed from the analyses, landraces revealed the existence of 10 genetic clusters (termed Q1–Q10) as highlighted by the Evanno method (Evanno *et al*., 2005) (Figure S3). This indicates that grouping by the structure analysis does not completely reflect grouping based on the geographical origin of collections. Exact geographical coordinates were available for a subset of 98 landraces collected from 15 of 16 regions sampled (Table S1). When projected onto a map, the sampling points revealed that the landraces are grown in a highly variable set of environments ranging in altitude between 1522 and 3163 masl (Figure S4).

**Figure 3:**
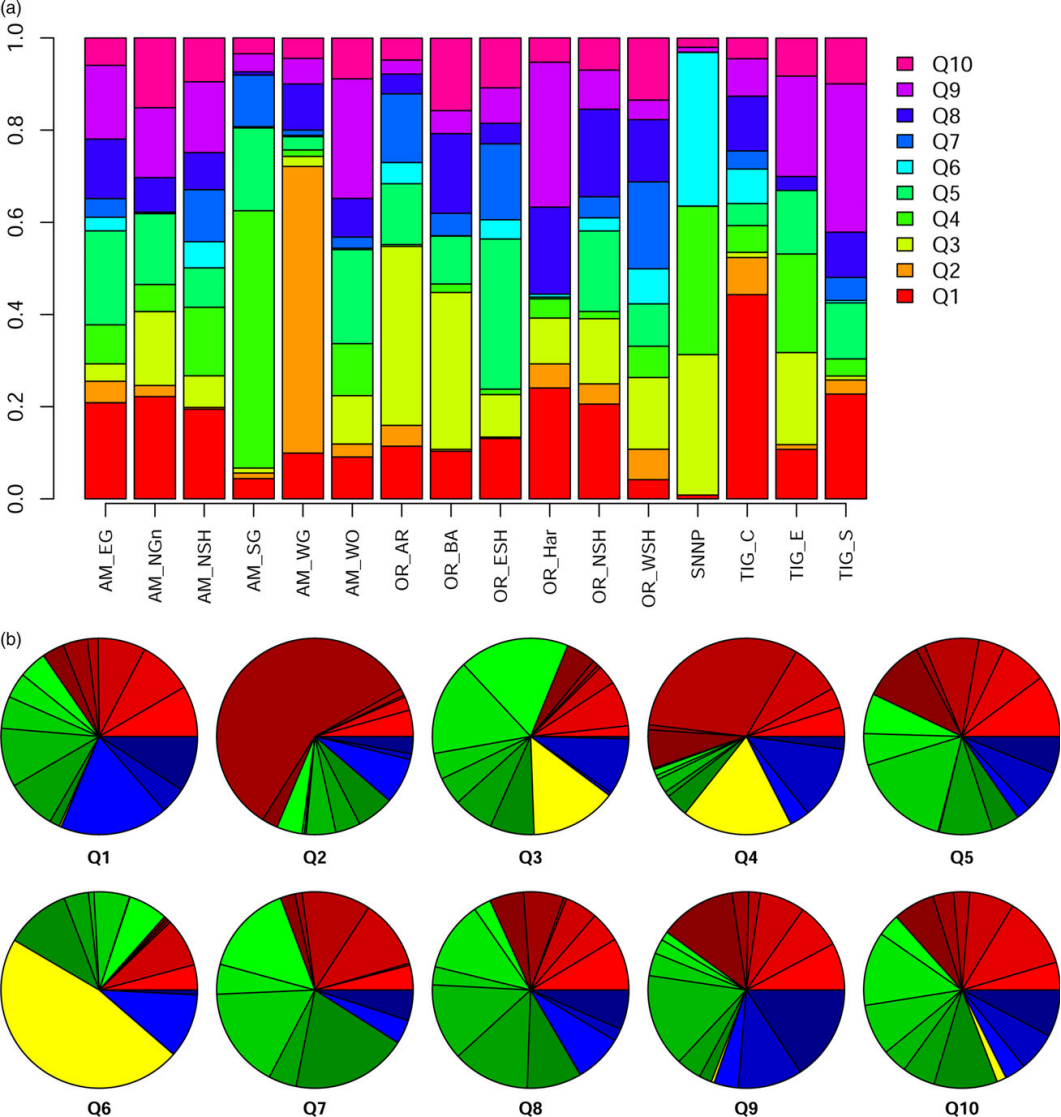
The Bayesian structure analysis showed the presence of 10 cryptic genetic clusters (Q1–Q10) within the landraces collection. (a) Cluster composition of the 16 sampling areas. While balanced overall, some districts have a peculiar cluster composition. (b) Spatial distribution of the 10 cryptic genetic clusters. The macro areas of Amhara, Oromia, Tigray and Southern Nations, Nationalities and Peoples are depicted in red, blue, green and yellow, respectively. Different shades of each colour indicate different subregions.

Despite clearly distinct sampling points, individuals are widely admixed, and very few ‘pure lines’ can be found (Figure S5). The molecular clustering of individuals does not reflect their geography of origin, showing that a substantial exchange of germplasm has occurred. We evaluated the relative contribution of each cryptic genetic cluster to sampling areas (Figure 3a). Even excluding the poorly sampled areas, which likely suffer significant sampling bias, some still show a significant degree of uniformity and uniqueness. This is the case of Amhara South Gonder (AM_SG) and Amhara West Gonder (AM_WG). Although close to each other, these two regions are mostly composed of Q4 and Q2 clusters, respectively. We surveyed the distribution of each genetic cluster by breaking down its contribution into different sampling areas (Figure 3b). These data are easier to interpret by collapsing sampling areas in the four wider regions of Amhara, Oromia, Tigray and Southern Nations, Nationalities and Peoples. The sole clusters with a balanced representation in the first three areas (the most sampled) are Q1 and Q9. Clusters Q5, Q7, Q8 and Q10 are also evenly distributed, even if in a lesser degree. Cluster Q2 seems typical of AM_WG. Cluster Q3 is prevalently distributed in Oromia, while cluster Q4 is prevalent in the Amhara region. Cluster Q6 is only found in one of the three samples collected in Southern Nations, Nationalities and Peoples region (Figure S5).

### Genome‐specific diversity and linkage disequilibrium (LD)

Markers with a map position (20 326 SNPs) (Maccaferri *et al*., 2015) covered a total length of 2562 cM (average marker density 0.13 SNP/cM). Genomes A and B did not show relevant diversity, indicating that they have followed similar evolutionary histories in Ethiopian landraces and improved varieties. The geodesic difference (Billera *et al*., 2001) between two genome‐specific trees scaled for genome A diversity is quantified as 17% and is mainly concentrated at the base of the trees (Figure S6). While the general features of the trees are maintained, some differences emerge in the landraces group.

**Figure 4:**
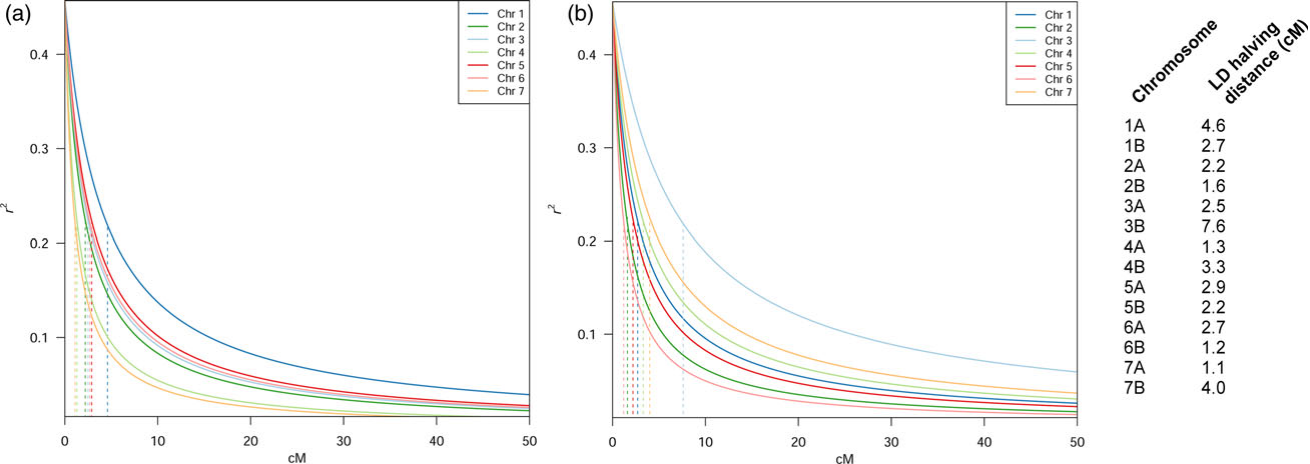
Linkage disequilibrium (LD) decay pattern according to the Hill and Weir function, with cM values on the *x*‐axis and *r* ^2^ values on the *y*‐axis. Per‐chromosome LD halving distance reported aside. (a) Chromosome‐specific LD decay on genome A. Each chromosome decay is depicted according to the colour shown in legend. Vertical broken lines report chromosome‐specific LD halving distance, in matching colours. Chromosome 1A has the slowest decay in the set. (b) Genome B specific LD decay. Each chromosome decay is depicted according to the colour shown in legend. Vertical broken lines report chromosome‐specific LD halving distance, in matching colours. Chromosome 3B has a notably slower decay than the others.

Linkage disequilibrium decays relatively fast in the collection (Figure 4). Homoeologous chromosomes show different LD decay patterns, as reported by the LD halving distance: chromosomes (Chr) 3, 4 and 7 show a much slower decay in genome B. The LD halving distance in the homoeologous pair of Chr 3 varies from 2.5 cM in genome A to 7.6 cM in genome B. Conversely, LD decay on Chr 1 is much faster on genome B than genome A, with an LD halving distance moving from 2.7 to 4.6 cM. Local pattern of LD confirms differences among the two genomes. Linkage blocks may be seen at different genetic positions in each homoeologous pair (Figure S7). This suggests a slight differentiation between the two genomes.

### Phenotypic diversity and GWA study

The analysis of variance revealed a highly significant (*P* < 0.001) variability due to the genotypes for all traits (Table 1). For combined data over locations, the broad sense heritability estimated for the traits ranged from 0.25 for NET to 0.89 for DB. The heritability estimate for GY and for its components was >0.5. The distribution of the estimated values based on restricted maximum likelihood (REML) of all phenotypic trait is reported in Figure S8.

**Table 1:** Mean values across genotypes and environments, range of variation for the genotypes, variance components for the genotypes (*V*_*g*_) and for genotype by environment interaction (*V*_*ge*_), and heritabilities (*h* ^2^) for the phenological traits days to booting (DB), days to flowering (DF) and days to maturity (DM), and for the agronomic traits number of effective tillers per plant (NET), plant height (PH), spike length (SPL), number of seeds per spike (SPS), grain yield (GY), biomass (Bm) and thousand‐grain weight (TGW)

	DB (d)	D (d)	DM (d)	PH (cm)	NET (n.)	SPL (cm)	SPS (n.)	Bm (t/ha)	GY (t/ha)	TGW (g)
Mean	73.3	83.2	132.7	95.5	4.67	7.21	31.4	7.37	2.38	39.6
Max	88.4	97.6	148.0	123.4	6.88	10.60	44.6	10.68	3.66	50.5
Min	63.0	73.5	116.0	62.0	3.00	4.99	24.0	3.91	0.85	25.9
*V*_*g*_	<0.0001^a^	<0.0001	<0.0001	<0.0001	<0.0005	<0.0001	<0.0001	<0.0001	<0.0001	<0.0001
*V*_*ge*_	<.0001	<.0001	<.0001	0.0081	0.4276	0.2976	0.0033	0.0386	0.0119	<.0001
*h* ^2^ (%)	89	89	69	85	25	73	67	57	64	84

a Significance of Wald test.

**Figure 5:**
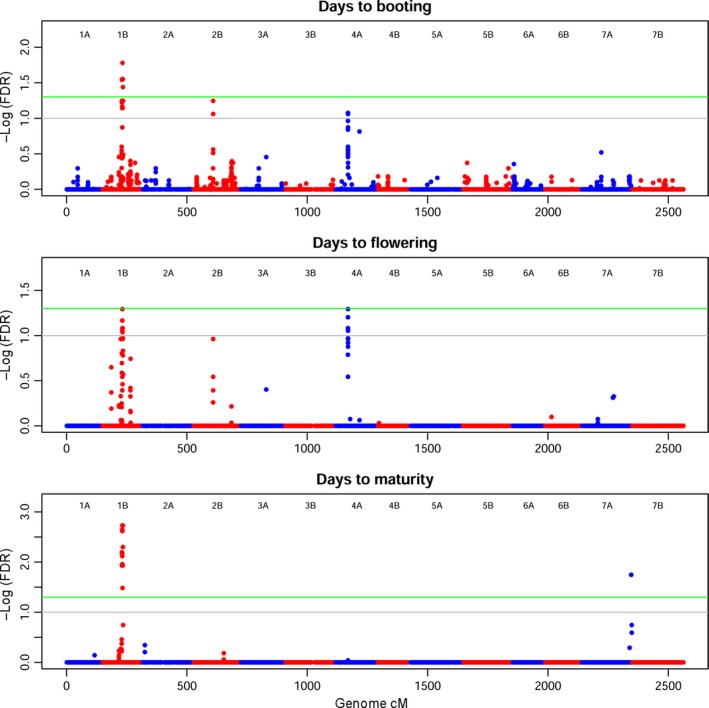
Manhattan plot for phenology traits. On the *x*‐axis, genetic position of markers in cM, considering the whole genome. Chromosomes are depicted alternately in blue (A genome) and red (B genome). On the *y*‐axis, the significance of the association tests reported as the negative logarithm of false discovery rate (FDR) values. Green line marks high significance (FDR 5%), grey line suggestive associations (FDR 10%). Panels are reported consequentially to flowering stages. Significant MTAs can be seen on Chr 1B, 2B, 4A and 7A.

Of the ten phenotypic traits analysed, many yielded a significant marker–trait association (MTA), even with the stringent FDR threshold applied (5%). All MTAs at FDR 5% are also shown in the LD pairwise plot in Figure S6. Spike length (SPL) and thousand‐grain weight (TGW) did not report any significant MTAs and thus are not reported. R/GAPIT was run using a compressed mixed linear model (CMLM) with different kinship algorithms, different compression models and dynamic counts of covariates for each trait. Quantile–quantile plots (QQ‐plots) resulting from the analyses were used as a guide to choose the model best fitting the data. VanRaden's method appeared superior in the data set under study and was used for all traits. The SUPER method yielded the most stable and strongest MTAs. QQ‐plots are shown in Figure S9, and all MTAs significant at FDR 10% are reported in Table S2. Phenological traits such as DB, DF and DM (Figure 5) confirmed the complex genetic background of the wheat flowering time. The best fit for phenological traits occurred with eight PCs as a covariate. These traits correlated as expected and share some common MTAs. The stringent threshold used left four peaks with varying effects throughout the flowering stages. The signal on Chr 1B is present from booting to maturity. The signal on Chr 2B is stronger in DB and then decreases in DF to disappear in DM. The MTA on Chr 4A is specific to booting and flowering, while the MTA on Chr 7A is specific to maturity.

**Figure 6:**
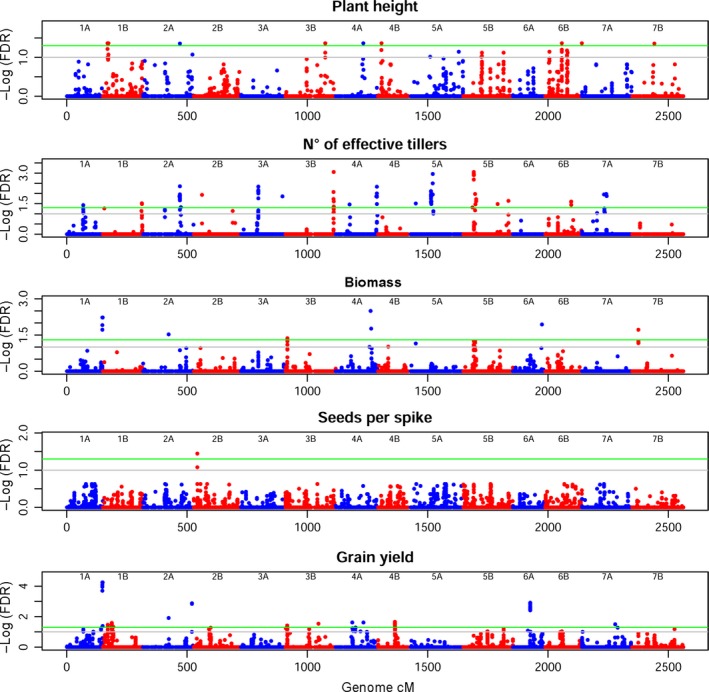
Manhattan plot for agronomic traits. On the *x*‐axis, genetic position of markers in cM, considering the whole genome. Chromosomes are depicted alternately in blue (A genome) and red (B genome). On the *y*‐axis, the significance of the association tests reported as the negative logarithm of false discovery rate (FDR) values. Green line marks high significance (FDR 5%), grey line suggestive associations (FDR 10%). Several significant and suggestive MTAs are present.

Agronomic traits are more elusive, as apparent from the Manhattan plots in Figure 6 and QQ‐plots in Figure S9. Plant height (PH) mapping was best when six PCs were considered. MTA reaching FDR 5% were eight, on Chr 1B, 2A, 3B, 4A, 4B and 6B. Notably, the statistical noise appears higher for this trait, suggesting a complex genetic control for PH. Bm revealed six MTAs. The highest significance was on Chr 4A, followed by Chr 1A and 6A, all much higher than FDR 5%. MTAs on Chr 2A, 3B and 7B also surpassed the high‐significance threshold. The highly complex trait of NET highlighted 16 highly significant MTAs. Six PCs as covariate revealed one relevant MTA on Chr 2B for seeds per spike (SPS). GY marked a peak of very high significance on Chr 1A. Other MTAs that surpassed the high‐significance threshold for this trait can be seen on Chr 2A, 3B, 4A, 4B, 6A and 7A.

## Discussion

### Diversity of Ethiopian durum wheat

Our Ethiopian durum wheat collection showed a high molecular and phenotypic diversity. The Ethiopian material generally showed increased failure rates and genomewide heterozygosity as compared to Mediterranean germplasms (Figure S1), even though mean heterozygosity remains very low in both panels (Figure 1). Relatively higher heterozygosity and failure rate in landraces are possibly contributed by additional polymorphisms in the probes, hampering efficient annealing on the chip and correct SNPs call. Failure rate and heterozygosity covary (Figure 1), reinforcing this hypothesis. Markers included on the array are preselected to be polymorphic in international wheat material and are unlikely to capture the full spectrum of new alleles in Ethiopian populations. A trade‐off exists between markers’ reliability and diversity captured. While other genotyping techniques such as reduced libraries sequencing (Rowe *et al*., 2011) would permit the discovery of new alleles, the 90k array provides the most reliable, and mapped, SNPs marker available today on the durum wheat genome. The genotyping array does not fulfil Ethiopian wheat diversity: by this, it confirms the potential of the collection in including broad molecular variation yet to be mined. Further studies will benefit from the employment of both hybridization and sequencing methods to provide a through description of Ethiopian wheat molecular diversity.

Compared to the sample of Mediterranean durum wheat in a diversity analysis, Ethiopian material confirmed very different (Figure 2a). Although Ethiopian wheat uniqueness has been advocated by several scholars, no studies to date provided high‐density molecular support for this hypothesis. Early observations reported Ethiopian wheat germplasm to be peculiar for rust resistance, drought tolerance, early ripening and several structural traits (Porceddu *et al*., 1973). More recent studies confirmed the uniqueness of Ethiopian landraces in varying degrees (Alamerew *et al*., 2004; Haile *et al*., 2013; Teklu *et al*., 2006), although using smaller panels and reduced genotyping density. This degree of differentiation seems to be a common feature of Ethiopian crops and is reported on barley as well (Igartua *et al*., 2013). The most significant PC in our PCA clearly separates improved wheat lines and Mediterranean material from landraces (Figure 2a). This analysis highlights two important points: Ethiopian germplasm poorly overlaps with Mediterranean durum wheat and Ethiopian landraces collect a much higher molecular variation (spread across PC2) than Mediterranean accessions. Durum wheat has been cultivated in Ethiopia for thousands of years, with little connections with wheat from outside the country, thus developing a unique set of characteristics (Smartt and Simmonds, 1995; Tsegaye and Berg, 2007a; Vavilov, 1992). The PCA failed to compact much variance in the first PC axes (Figure 2b; 50% cumulative variance only at PC13). This suggests that the landrace panel has a poor structure and is composed of highly admixed lineages. The diversity is also high, as reported by more than 30 000 polymorphic SNPs scored in the Ethiopian durum wheat set. To contextualize this diversity, this is comparable to the number reported on the same chip for an elite hexaploid wheat population, in that case also contributed by the D genome (Sukumaran *et al*., 2015). Although limited in numbers, the Mediterranean durum wheat here included is still representative of the wheat currently cultivated in most of the corresponding regions (Table S1): its molecular diversity as detected by the array is very limited as compared to Ethiopian landraces (Figure 2).

Genetic diversity in worldwide durum wheat has been shown to be partially depleted by breeding (Ren *et al*., 2013), and Ethiopian material diversity confirms this general picture. Some Ethiopian genotypes, however, cluster with Mediterranean material in the PCA space (Figure 2), notably improved varieties introduced to local farming systems since 1967 (http://wheatatlas.org/country/varieties/ETH/0). This is expected as most of these materials were imported from international breeding efforts that to date have made little to no use of Ethiopian diversity. We did not find any association between the year of introduction and the degree of similarity shown by landraces and improved varieties (Figure S2). This suggests the absence of a trend towards the progressive introduction of landraces in released material or *vice versa*. Some improved varieties and landraces are exceptions to this general trend, some clustering in the bulk of the others (Figure S2). Although some local breeding efforts might well have introduced international diversity in Ethiopian landraces and the other way around, this admixing is possibly due to contaminations at the seedbank level.

The structure analysis (Figure 3) confirmed the general admixture of samples reported by the PCA and showed that the number of genetic clusters was lower than that of the locations where the materials were sampled. Note that the panel under study does not entirely fit a natural population. The expectancy of isolation by distance (Wright, 1943) does not stand, as this wheat panel is historically and currently exchanged through an informal seed system involving regional and countrywide farming communities (Seboka and Deressa, 1999). Although Structure is best used under the Hardy–Weinberg equilibrium (HWE), it may provide a consistent overview of the genetic diversity of panels breaking HWE and linkage equilibrium assumptions (Rodríguez‐Ramilo *et al*., 2009), as well as under varying dispersal regimes (Evanno *et al*., 2005). We identified a clear peak for the 10 cluster hypothesis (Figure S4) and a high rate of admixture throughout (Figure 3). Landraces are widely admixed, both in genetic and geographical terms (Figures S2 and S3). Still, some sampling areas present a marked enrichment in specific genetic clusters (Figure 3): South Gonder and West Gojam, in Amhara region, show an extensive and characterizing the presence of Q4 and Q2, respectively (Figure S5). This may be the consequence of the local preferences of farmers towards a given lineage, driven by local climatic conditions or socio‐economic forces. Note that the South Gonder samples were collected at more than 3000 masl, while West Gojam samples were collected 1000 m lower. More studies are needed to evaluate the relative importance of the environment in the spatial distribution of landraces, although other factors are likely to affect genetic cluster distribution, notably seed availability, linguistic barriers, and even the availability of infrastructures permitting seed circulation.

The similar degree of diversity shown by the A and B genomes in the landrace panel (Figure S6) suggests a common evolution of AABB wheat in Ethiopia. However, the high degree of genetic diversity shown by the Ethiopian wheat suggests that the evolutionary history of wheat in East Africa could be re‐evaluated by molecular means. Understanding exactly when durum wheat was introduced into Ethiopia and the corresponding separation from Mediterranean durum wheat is a matter of great interest both from a breeding and a historical standpoint, and further studies will shed light on this topic.

### Usefulness of landrace panels in durum wheat GWA studies

Our Ethiopian durum wheat panel represents the first attempt to provide a thorough characterization of new molecular diversity that might be of use in durum wheat breeding efforts. As wheat is threatened by new pathogens, climate change and the scarcity of resources (Anwar *et al*., 2007; Challinor *et al*., 2014; Juroszek and von Tiedemann, 2013), a new injection of allelic variation may be needed to overcome current breeding *plateaus* (Ceccarelli *et al*., 2010). LD in the mapping panel showed a varied decay in the homoeologous genomes (Figure 4). This might be partly due to the different sets of markers used, in a slightly lower number in genome B. If this was the case, however, we should expect a similar behaviour for all chromosomes, but there is no such behaviour. The different rates of LD decay may report different evolutionary forces at play in homoeologous pairs, by either selection for the presence of QTL or genetic drift. The general picture emerging is that of a relatively rapid LD decay, confirming the great diversity in Ethiopian durum wheat. Note that some linkage blocks show a peculiar pattern, alternating markers in high LD to markers at low to null LD (Figure S7). Regions of identity by descent (IBD) at the establishment of the panel may alter long‐range LD patterns, inflating linkage measures because of low allelic diversity. IBD and population structure may contribute in increasing LD measures and long‐range LD in durum wheat elite line collections (Maccaferri *et al*., 2005), as well as in determining focalized regions of substantially high LD (Somers *et al*., 2007). The aforementioned studies used a much lower number of markers and genotypes but, even if at a coarser resolution, provided a similar picture to that identified in our panel. Ethiopian durum wheat accessions also reported long‐range and even interchromosomal high LD with only 28 microsatellite loci analysed (Mondini *et al*., 2010). This is also possibly due to IBD, selection, or drift, even though the broader diversity of Ethiopian wheat suggests another possible explanation. As the width of populations and the level of admixture increases (Figure 3), in fact, evolutionary forces other than genetic drift may affect LD patterns. The high number of genotypes and markers analysed in our study permits us to hypothesize that long‐range LD may be due to altered map positions of SNP markers in the Ethiopian sample. The durum wheat genetic map in use was developed on international germplasm (Maccaferri *et al*., 2015), which may show structural variation with Ethiopian material. Structural variation is a diffused phenomenon in crop genomes and was perhaps best characterized in maize (Chia *et al*., 2012). Studies on structural variation in wheat are still limited to single loci (Nishida *et al*., 2013); however, it is likely that the wheat genome (with more than 80% in repeated DNA) also shows extensive structural variation and copy number variation. A better characterization of our panel will soon be made possible by the construction of a specific genetic map on Ethiopian durum wheat material. We developed a nested association mapping (NAM) population (McMullen *et al*., 2009) derived from this same material, built by sampling 50 highly diverse durum wheat landraces which were crossed with a recurrent Ethiopian improved cultivar. This tool will lead to the production of a high‐density genetic map of Ethiopian durum wheat, which together with the current durum wheat map will permit a finer characterization of genomic rearrangements in durum wheat.

The speed of LD decay is a relevant trait in an efficient mapping panel, as it determines the capacity to identify MTAs. The broad amount of diversity in our mapping panel leads to a rather fast LD decay for a typically autogamous species, dropping below *r* ^2^ 0.2 at around 5 cM on average (Figure 4). This is particularly important in conditions of less than optimal marker density, as is the case of wheat. Although the identification of causal variants of QTL in wheat might be still out of reach, GWA studies on Ethiopian material may help to (i) report the presence of yet unknown causative regions and (ii) guide marker‐assisted breeding programmes to introduce new allelic variation in elite wheat germplasm.

We identified several genomic regions associated with the traits under study. The high threshold employed (FDR 5%) was often surpassed by several orders of magnitude in the case of loci with high effects. For some traits, the low heritability and the complex nature of the trait (with likely multiple causative loci each with low effects) in a background of relatively rapid LD decay with a low SNP density hampered the discovery of signals at FDR 5%. The mapping method we used successfully lowered the inflation of *P*‐values; however, for the most complex traits still showed some inflation (Figure S9). The conservative threshold employed enabled us to call several significant MTAs with good reliability for most of the traits analysed, some already reported, but the majority still undiscovered.

Phenology in the Ethiopian panel reported four major loci with alternating effects throughout the flowering process, from DB to DF and finally DM (Figures 5 and S7). Three signals on Chr 1B (79.6–84.5 cM), 2B (80.6 cM) and 4A (50.8–51.3 cM) are clear in DB; then, in DF, the signal on Chr 2B decreases, while on Chr 4A increases above the FDR 5% threshold. DM are mostly contributed by Chr 1B, while a new MTA appears on the distal portion of Chr 7A (204.2 cM). A previous study using a different genetic map identified QTL involved in flowering on Chr 1B and 2B in a structured population of elite durum wheat (Sanna *et al*., 2014). The same study reported additional QTL on an intermediate map position on Chr 4B and in the distal portion of Chr 7B, while our study identified MTAs on similar regions of the homoeolog chromosomes 4A and 7A. Notably, ancient translocation events on chromosome groups 4 and 7 (as well as 5) were reported in several studies focusing on the collinearity of durum and bread wheat genetic map (Devos *et al*., 1995; Ma *et al*., 2013; Maccaferri *et al*., 2015). Thanks to its long separation from the international durum wheat pool, the construction of an Ethiopian durum wheat genetic map could help in shedding light on the evolutionary history of such events and identify chromosomal rearrangements. The MTAs on Chr 2B and 4A confirm those reported in similar map positions in a previous study (Maccaferri *et al*., 2008).

Several MTAs for agronomic traits were detected, including new loci. We identified several MTAs for PH, a trait of renowned complexity in wheat (Maccaferri *et al*., 2008; Zanke *et al*., 2014). At FDR 5%, we identified MTAs on Chr 1B (19.4 and 23.2 cM), 2A (154.6 cM), 3B (166 cM), 4A (114.6 cM), 4B (14.4 cM), 6B (71.9 and 155.1 cM) and 7B (92 cM). Only a few of our highest significance MTAs may be put in topological relation with *Rht‐1* like genes, a gene family of major importance in the determination of dwarfism in elite wheat (Pearce *et al*., 2011; Tan *et al*., 2013). We found an MTA on Chr 3B, where *Rht5* had already been reported, but at a different map position. The map position reported for *GA20ox1* and *KAO‐B1* makes them candidates for our MTA on Chr 4A. *Rht13* co‐localizes with the MTA on Chr 7B (Zanke *et al*., 2014). It should be noted that the majority of studies conducted up to now have focused on bread wheat, and that durum wheat might show a different composition for these complex traits. A QTL study on durum wheat (Maccaferri *et al*., 2008) identified a PH QTL on proximal portions of Chr 1B and Chr 4B, close to our MTAs. Maccaferri *et al*. also reported a QTL for PH on Chr 2B and 7A, on map positions similar to the MTAs we found on Chr 2A and 7B.

The NET and Bm traits have been poorly studied in durum wheat, as primitive traits repressed in elite cultivars. Undetermined tillering increases shading of flag leaves and reduces the harvest index (Hay, 1995; Richards, 1988), thus being selected against during breeding. The tiller inhibitor gene *Tin* has been reported on the short arm of Chr 1A in wheat (Spielmeyer and Richards, 2004). Bm is also a generally undesirable trait, as inversely related to yield in determining the harvest index. However, both these traits are valuable in Ethiopia, as straws are used for livestock feed and for house thatching. The several signals we identified for these two traits highlight that Ethiopian material is an important starting point to examine the molecular mechanisms behind wheat architecture determination.

The sole significant signal for SPS is placed on Chr 2B at 14.5 cM. Although this trait can be considered as a yield component, this MTA is not matched by the grain yield (GY) scan. The GY reports many MTAs, a few of which are very significant and expectedly pleiotropic on Bm. Chr 1A (148.3 cM) and 6A (71.8 cM) show the strongest signals. No matches in the literature were found for these loci, requiring further analyses. GY QTL on durum wheat has been reported on Chr 2B, 3B and 7B (Maccaferri *et al*., 2008): of these, the proximal QTL on Chr 3B was confirmed in our study at FDR 5%.

### Perspectives

We have shown how Ethiopian landraces may represent an important source of wheat variation. We are in the process of redistributing the most relevant landrace material included in this study to Ethiopian small farmers within a crowdsourcing approach (van Etten, 2011), directly involving the farmers as selectors and beneficiaries at once. Although not targeted by this study, Ethiopian hexaploid wheat could be similarly differentiated from international material. The Ethiopian landrace panel used here has the advantage of being modular, thus allowing for the addition of new genotypes and new genotyping efforts. Enhancing and promoting Ethiopian germplasm in a breeding perspective would also result in more efficient landrace conservation (Tsegaye and Berg, 2007b).

New markers and sequences will permit a finer genomic characterization of these and other genotypes in the near future, permitting a further characterization of the discovered MTAs. We plan to use denser molecular markers based on low‐depth sequencing, permitting us to address and eventually identify the causal variants in the genomic background of this material. One complementary approach providing more power while considering elevated diversity is to build multiparental mapping populations by harvesting broad allelic variations in a structured panel. The use of our Ethiopian durum wheat NAM, currently being characterized, will allow for a better definition of the QTL signals evidenced in this study and, by the accumulation of phenotypic measures, will lead to the discovery of important durum wheat loci for international breeding efforts.

## Experimental procedures

### Diversity panel assembly

The plant material was collected among Ethiopian wheat accessions conserved at the Ethiopian Biodiversity Institute (EBI; www.ibc.gov.et). Passport data were a necessary requirement to consider accessions, as we aimed to gather a panel representative of durum wheat diversity from throughout Ethiopian wheat‐growing areas. The panel was composed of accessions recorded as durum and/or tetraploid wheat collected in 16 different regions of Ethiopia, for a total of 287 accessions. Twenty‐four improved varieties, that is varieties resulting from documented breeding efforts and registered for cultivation in Ethiopia, were added to the collection from other providers (Table S1). The full panel of 311 Ethiopian varieties (5 g of seeds each) was multiplied during the off‐season of 2011 at Mekelle University research station, followed by 2011 main season to check uniformity of accessions. When two or three types were present in the same accession, it was split accordingly and identified types were marked with a letter (A, B and C), stored separately and considered as independent accessions. Even after the check for homogeneity, landraces were expected to show a certain degree of internal heterogeneity. We decided to produce and study a standard genotype for each, using a spike derived from a single plant per landrace as a seed donor for growing the seedlings that provided the plant material for subsequent DNA extraction and eventually for field trials. Therefore, when we refer to the genotypic characterization of a specific landrace or variety, we are in fact referring to a standard reference genotype that is kept distinct from the original landrace. The panel of Ethiopian wheat was sided with a collection of 38 Mediterranean durum wheat acting as out‐group for diversity analyses, totalling 349 samples.

The original Ethiopian landraces accessions are available upon request from the EBI, focal point for CBD and ITPGRFA regulating exchange of germplasm. Access to the material is subject to domestic legislation in line with the international conventions and treaty ratified by Ethiopia (CBD and ITPGRFA). The diversity panel here described is also available at EBI under the same regulation: accessions used in this study are marked with a DP (for diversity panel), followed by the original EBI naming (Table S1). Ethiopian improved varieties are available at Sirinka Agricultural Research Center (P.O. Box 74, Sirinka (Woldia), Ethiopia). Mediterranean material is available at Scuola Superiore Sant'Anna by contacting the corresponding author.

### DNA extraction and genotyping

Genomic DNA was extracted from fresh leaves pooled from five‐one‐month‐old seedlings for each of the accessions analysed. Seedlings were pooled to provide a full representation of each standard landrace genotype. Sampling and pooling of five seedlings permits to detect heterozygosity at a given diploid locus with a binomial probability of 0.9375. The GenElute Plant Genomic DNA Miniprep Kit (Sigma‐Aldrich, St Louis, MO) was used following the manufacturer's directions at the Mekelle University Molecular and Biotechnology Laboratory. Genomic DNA was checked for quantity and quality by electrophoresis on 1% agarose gel and Nanodrop 2000 (Thermo Fisher Scientific Inc., Waltham, MA). Genotyping was performed on the Infinium 90k wheat chip (Wang *et al*., 2014b) at TraitGenetics GmbH (Gatersleben, Germany). SNPs were called using the tetraploid wheat pipeline in GenomeStudio V11 as described in Wang *et al*. (2014b). Genotyping data are available as a supplementary file, Table S3.

### Molecular diversity analyses

Two groups of wheat samples were considered for diversity analyses. The full data set, including Mediterranean samples as out‐group, was used for broader diversity analyses. The subset of Ethiopian material, including durum landraces and improved varieties, was subsequently considered. Polymorphism count, failure rate and heterozygosity were calculated with custom scripts in R. The broad genetic relationships among individuals were analysed with a PCA (Price *et al*., 2006) with R/SNPrelate (Zheng, 2013) considering all SNPs. A NJ phylogeny was also calculated on the full set of SNPs with R/ape 3.1 (Paradis *et al*., 2004).

Structure 2.1 (Pritchard *et al*., 2000) was used to estimate the number of cryptic genetic clusters (*K*) in the data set and to estimate the membership probability of each genotype to the subpopulations. Structure was run with standard settings (1000 burn‐in and 10 000 effective sweeps), five replicates for each *K* from *K* = 1 to *K* = 20. The Evanno method was used to identify the most probable number of clusters with Structure harvester (Earl and vonHoldt, 2012). As the separation between Ethiopian landraces and improved varieties masked any other clustering, the analysis was rerun on the landraces data set only. *R* was used to collapse runs from the best *K*, and count the cluster assignation across the geographical areas of origin of Ethiopian landraces. Sampling point coordinates in WGS84 were obtained by EBI, when available. Sampling points were projected onto a map of Ethiopia in QGIS 2.6.1 Brighton (Quantum GIS Development Team, 2012) on the background of altitude values obtained from WorldClim 1.4 (Hijmans *et al*., 2005).

### Map‐dependent analyses

Single nucleotide polymorphisms with a map position on the durum wheat genome (Maccaferri *et al*., 2015) were used for map‐dependent analyses. We surveyed the genome‐specific diversity in the A and B genomes using R/distory (Chakerian and Holmes, 2013) to calculate the topological convergence of NJ trees calculated on the A and B genome separately.

Linkage disequilibrium was calculated on all chromosomes with R/LDheatmap (Shin *et al*., 2006). The *r* ^2^ measure was selected as it accounts for varying minor allele frequencies. LD measures between all pairs of SNPs on each chromosome were collapsed in a matrix reporting intermarker distances and used to calculate the LD decay in 50 cM with a custom script. The Hill and Weir equation (Hill and Weir, 1988) was used to estimate LD as a function of genetic distance (Marroni *et al*., 2011). R/LDheatmap was used to produce LD heatmaps for each chromosome.

### Field experiments and phenotyping

Four field trials (hereafter considered as environments) were carried out in two growing seasons (2012/2013 and 2013/2014) and at two locations, in Geregera in the Meket district (Wollo, Amhara region, 11°40′N/38°52′E) and in the Hagreselam district at Melfa village (Tigray region, 13°39′N/39°10′E), under field conditions (Figure S3). Both sites are often used as test fields for the two regions and present different climatic and edaphic conditions that vary in altitude, rainfall, temperature, soil type and other agro‐climatic factors. Accessions were sown in four rows 2.5 m long at a seed rate of 100 kg/ha in two replications using 20 × 20 partial lattice designs. Full doses of P (100 kg/ha DAP) and 1/2 of N (50 kg/ha urea) fertilizers were applied during sowing. Additional 50 kg/ha of urea was applied at the beginning of the tillering stage in both sites. Weeds were controlled manually. Ten traits, accounting for yield, yield components and phenology, were investigated. The three phenologic traits, days to 50% booting (DB), days to 50% flowering (DF) and days to 75% maturity (DM), were recorded for whole plots following Zadock's growth stages. Three randomly selected plants per plot were considered to measure (i) number of effective/productive tillers per plant (NET; measured as number of tillers producing spikes per plant), PH (in cm), SPL (measured as the distance between the pedicle base and the tip of the spike excluding awns, in cm) and number of SPS. The full plot was used to measure GY (measured as grams of grain produced per plot, converted in t/ha), above‐ground biomass (Bm; measured as dry weight of the above‐ground harvested Bm grams per plot, in t/ha) and TGW (measured as the weight of 1000 kernels, in grams). Grain moisture content was measured using SATAKE Digital Grain Moisture Meter (SS – 7) and used to adjust GY and TGW to standard moisture content of 12.5%.

### Phenotypic data analysis

Phenotypic data analyses were conducted according to a mixed model including genotype, location, genotype x location interaction, replication and block effects. Variance components were estimated according to the following model:

(1) $$y_{ijkl}=\mu +g_{i}+e_{j}+r_{jk}+b_{kjl}+\varepsilon_{ijkl}$$

where *y*_*ijkl*_ is the observation for genotype *i* at location *j* in replication k in block l. In the model, μ is the overall mean, *g*_*i*_ the effect of the inbred line *i*,* e*_*j*_ the effect of environment *j*,* g*_*eij*_ the interaction between inbred line *i* within environment *j*,* r*_*jk*_ the effect of replication *k* within environment *j*,* b*_*kl*_ the effect of incomplete block *l* within replication *k*, and ε_*ijkl*_ the residual. All effects in Eq. (1) except μ were considered as random to estimate variance components and were computed by REML. A Wald test (Wald, 1943) was used to test whether variances were significantly greater than 0. Heritabilities (*h* ^2^) were calculated on an entry‐mean basis, across the four environments (*n*) and the two replications (*r*) per environment, as:

(2) $$h^{2}=\sigma_{g}^{2}/\left(\sigma_{g}^{2}+\sigma_{g*e}^{2}/n+\sigma^{2}/nr\right)$$

where $\sigma_{g}^{2}$ is the genotypic variance, $\sigma_{ge}^{2}$ is the genotype by environment interaction variance, and σ^2^ is the residual error variance (Falconer and Mackay, 1996). For the calculation of genotypes adjusted means, best linear unbiased estimates were computed by considering μ, and *g*_*i*_ in Eq. (1) as fixed effects while the remaining effects were considered as random. Computations were performed using PROC MIXED in SAS (SAS Institute, Cary, NC).

### Genomewide association study

The R package Genome Association and Prediction Integrated Tools (GAPIT) (Lipka *et al*., 2012) was used to perform a CMLM onto mapped SNPs in the Ethiopian data set. Minor allele frequency was filtered at 5%. The structure within the panel was estimated with a PCA on the mapping panel iteratively considering from one to 12 PCs as covariates. Kinship was iteratively calculated with the VanRaden (2008), Loiselle *et al*. (1995) and EMMA methods (Kang *et al*., 2008). The SUPER method (Wang *et al*., 2014a) within GAPIT was used to calculate associations. The best fit of the model was evaluated on the QQ‐plots generated by the model. Nominal test significance was corrected for multiple testing by considering a false discovery rate (FDR) calculated on the basis of Storey's Q method (Dabney *et al*., 2013). Associations with an FDR below 5% were deemed highly significant, while all MTAs above FDR 10% are discussed when relevant.

## Supporting information


**Figure S1** Boxplots showing the performances of the array in different sub‐panels.

**Figure S2** NJ tree of the Ethiopian material.

**Figure S3** Altitudinal map of the area under study.

**Figure S4** Output for Evanno's ∆*K* when considering only Ethiopian landraces in a Structure analysis.

**Figure S5** District compositions in genetic clusters.

**Figure S6** Geodesic difference of NJ phylogenies considering genome‐specific diversity.

**Figure S7** LD heatmaps for each chromosome in separate panels.

**Figure S8** Histograms of the distribution of estimated phenotypic values.

**Figure S9** Quantile‐quantile plots (QQ‐plots) for GWA analysis as produced by R/GAPIT.

**Table S1** Complete information for the durum wheat samples included in this study.

**Table S2** Marker‐trait associations (MTAs) significant at FDR 10%.

**Table S3** Genotypic data used in this study.
