# Supplementary figures and images for: High‐density molecular characterization and association mapping in Ethiopian durum wheat landraces reveals high diversity and potential for wheat breeding
Source: Plant Biotechnol J. 2016 Feb 8;14(9):1800–12. doi: 10.1111/pbi.12538 (PMC5067613; doi:10.1111/pbi.12538)

**a** Heterozygosity

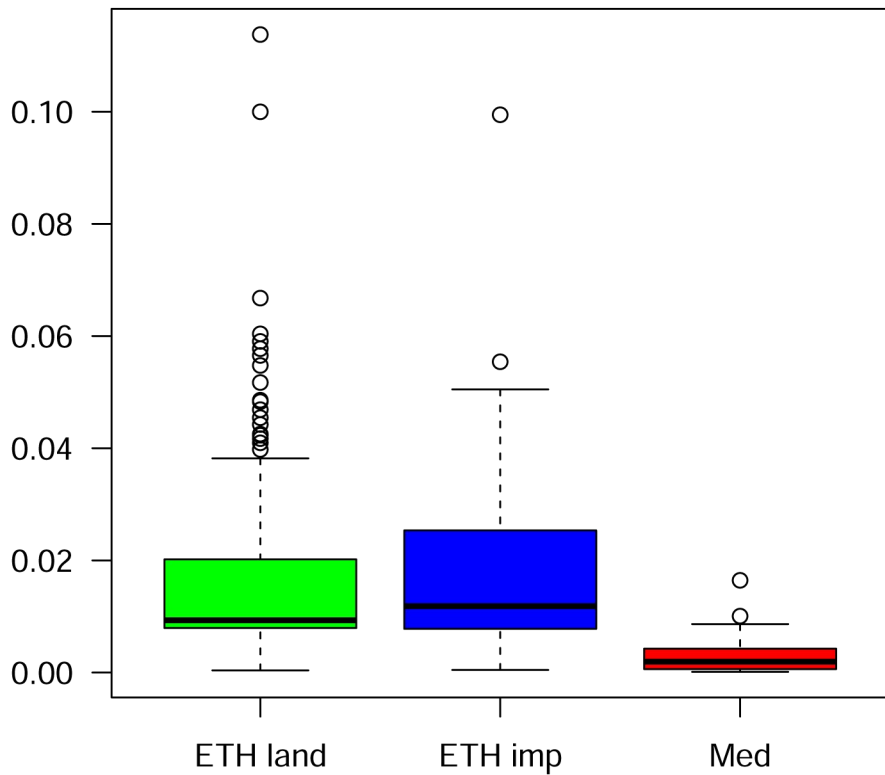

**b** Failure Rate

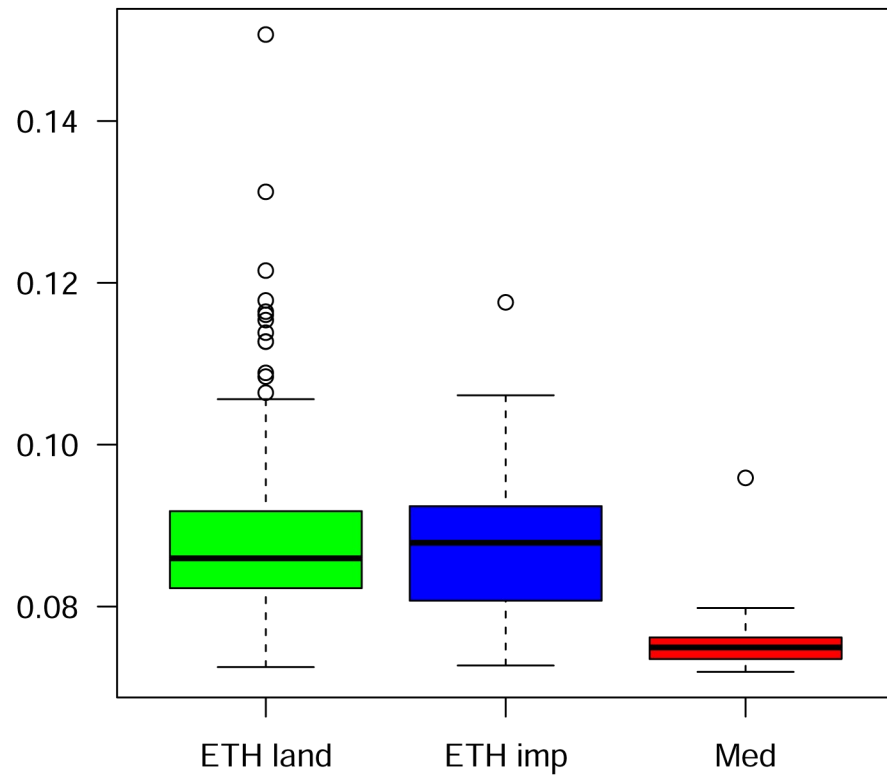

Supplement: Supplementary file 1 — Figure S1 Boxplots showing the performances of the array in different sub‐panels. [file PBI-14-1800-s006.pdf]

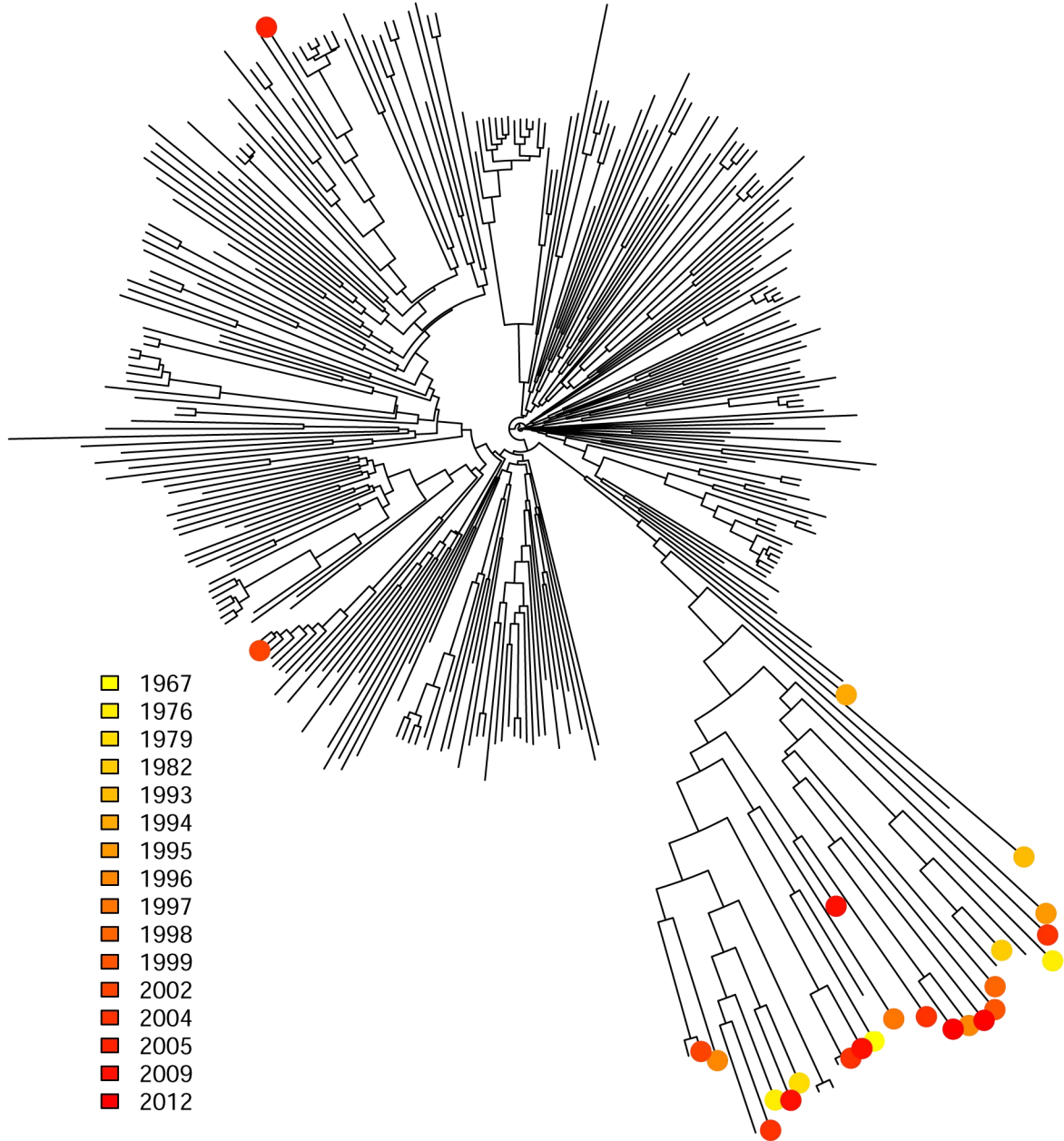

Supplement: Supplementary file 2 — Figure S2 NJ tree of the Ethiopian material. [file PBI-14-1800-s010.pdf]

## Legend

- Sampling points
- Experimental fields

Regional borders

Altitude (masl)

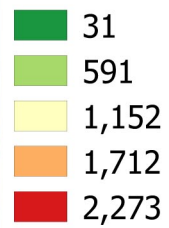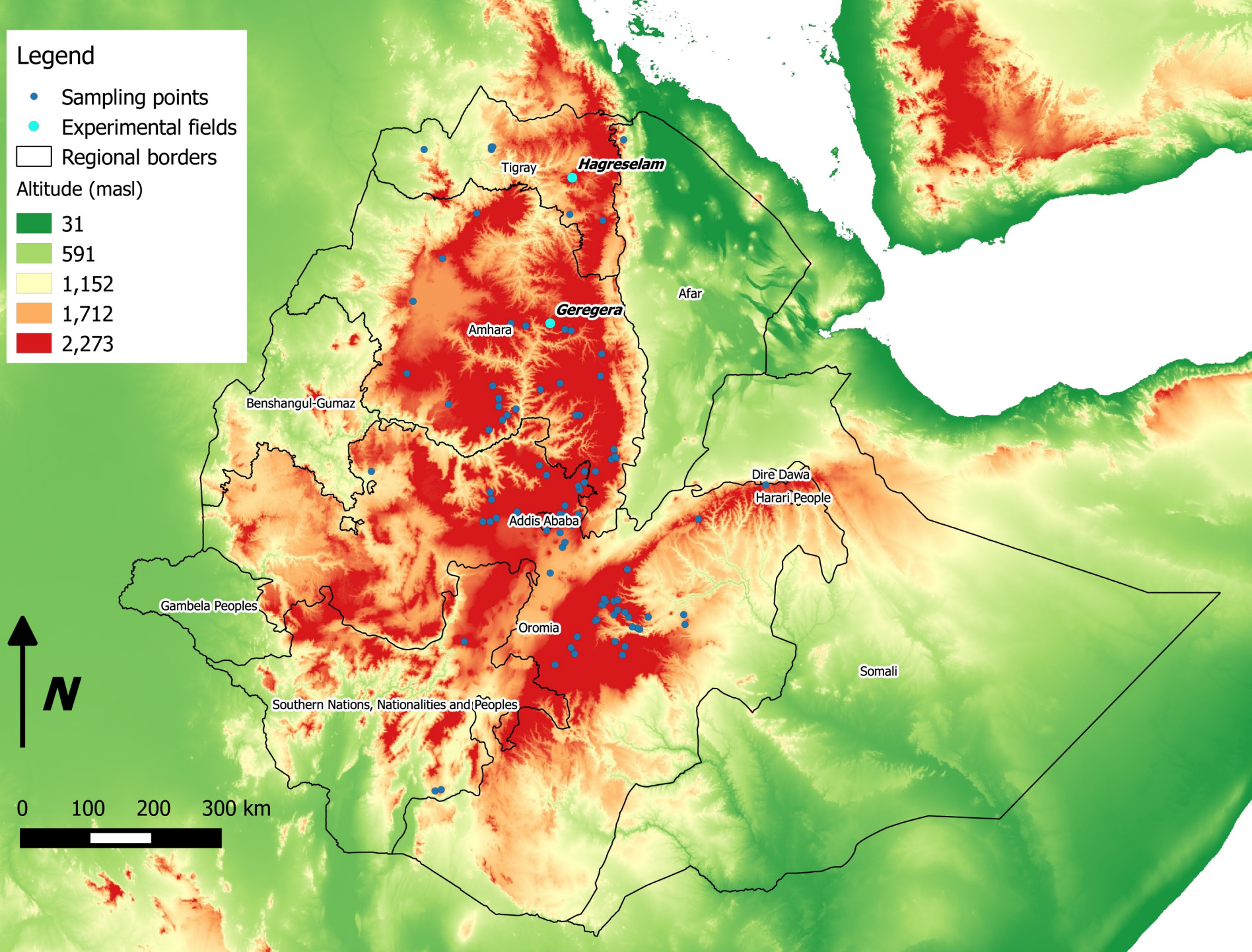

Supplement: Supplementary file 3 — Figure S3 Altitudinal map of the area under study. [file PBI-14-1800-s009.pdf]

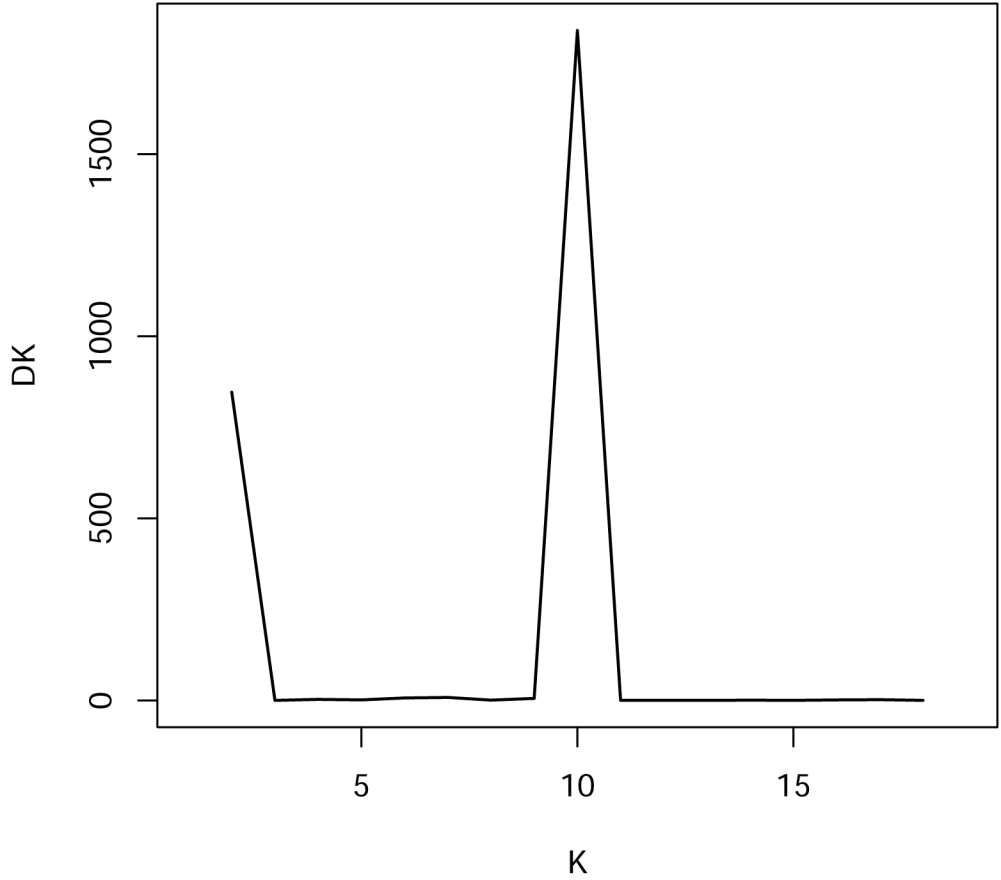

Supplement: Supplementary file 4 — Figure S4 Output for Evanno's ∆K when considering only Ethiopian landraces in a Structure analysis. [file PBI-14-1800-s011.pdf]

AM\_EG

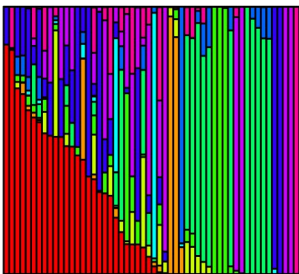

AM\_NGn

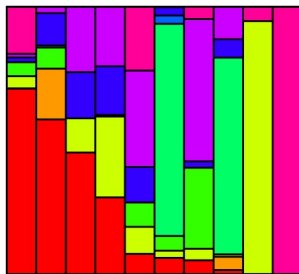

AM\_NSH

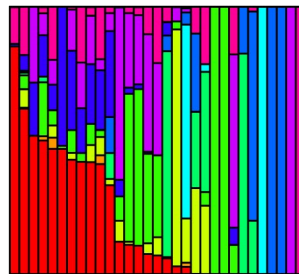

AM\_SG

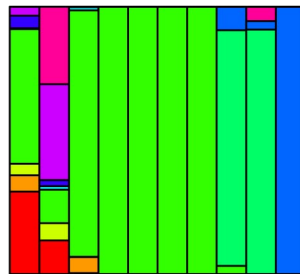

AM\_WG

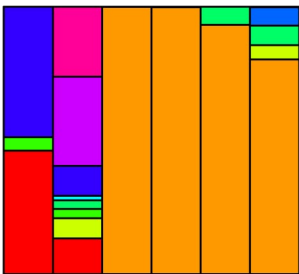

AM\_WO

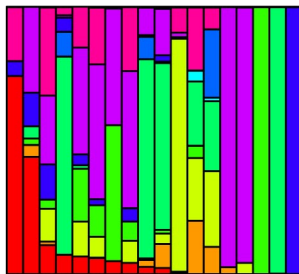

OR\_AR

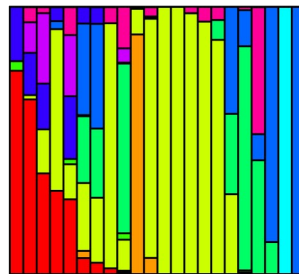

OR\_BA

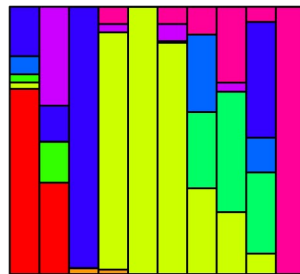

OR\_ESH

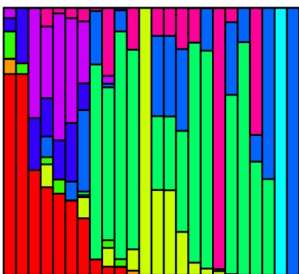

OR\_Har

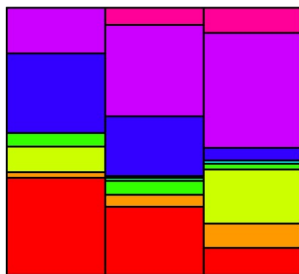

OR\_NSH

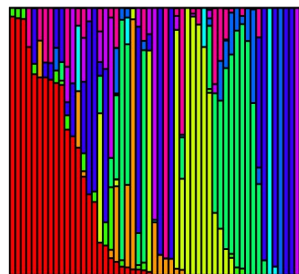

OR\_WSH

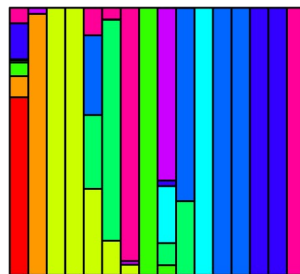

SNNP

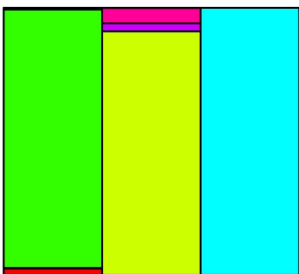

TIG\_C

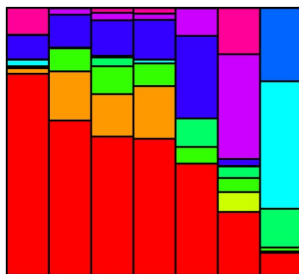

TIG\_E

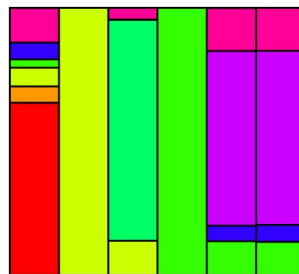

TIG\_S

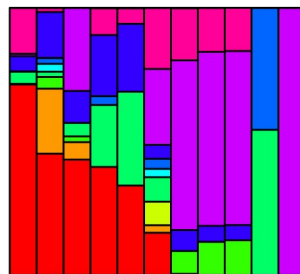

Supplement: Supplementary file 5 — Figure S5 District compositions in genetic clusters. [file PBI-14-1800-s001.pdf]

**a**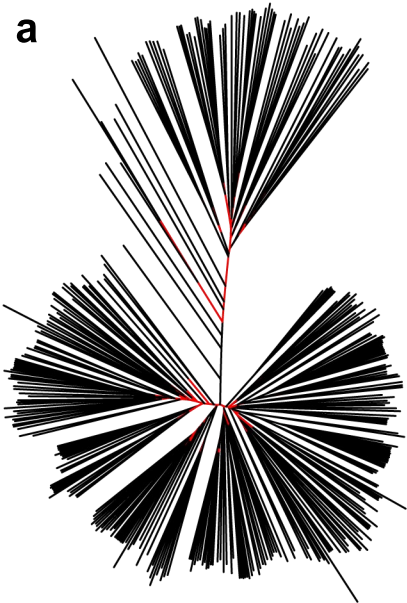**b**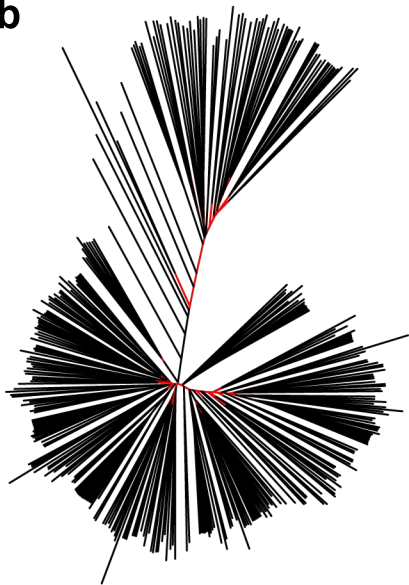

Supplement: Supplementary file 6 — Figure S6 Geodesic difference of NJ phylogenies considering genome‐specific diversity. [file PBI-14-1800-s002.pdf]

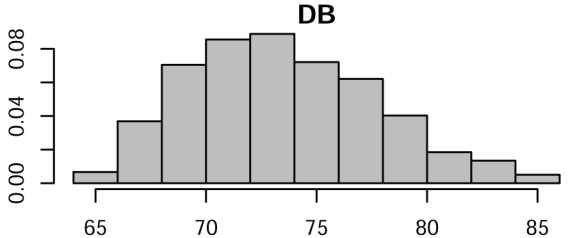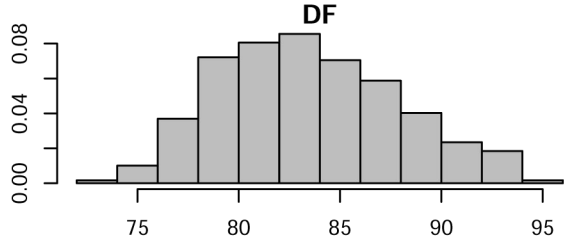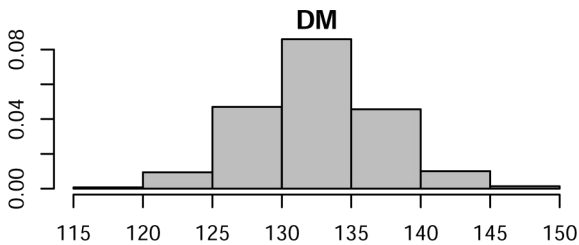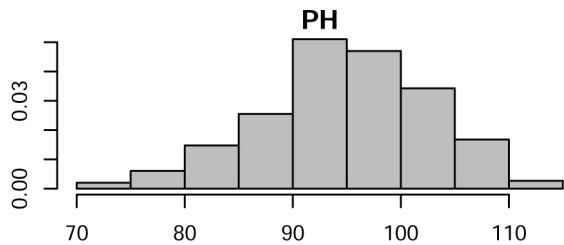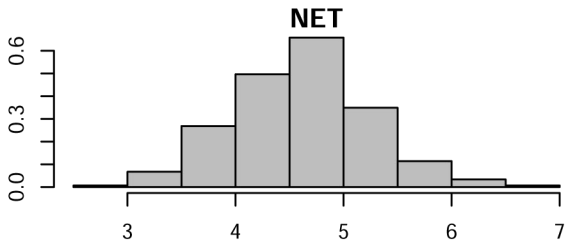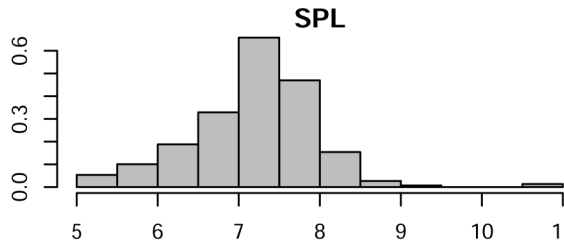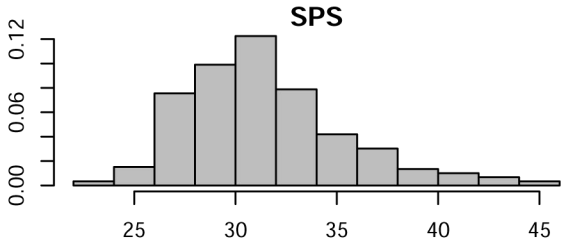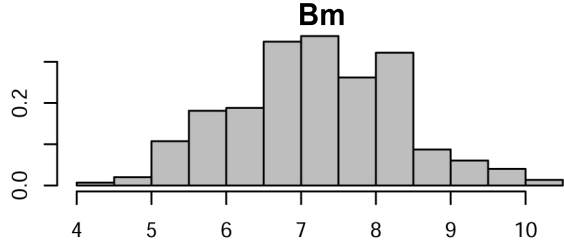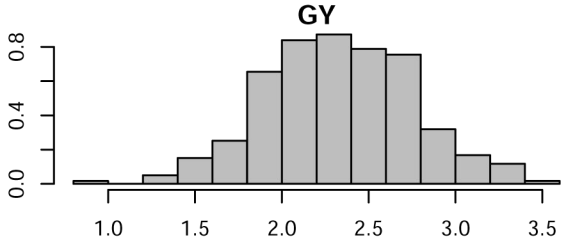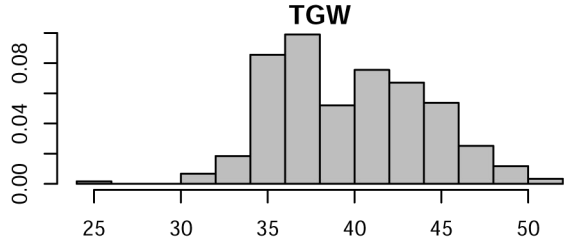

Supplement: Supplementary file 8 — Figure S8 Histograms of the distribution of estimated phenotypic values. [file PBI-14-1800-s004.pdf]
