# Supplementary material for: High‐density molecular characterization and association mapping in Ethiopian durum wheat landraces reveals high diversity and potential for wheat breeding
Source: Plant Biotechnol J. 2016 Feb 8;14(9):1800–12. doi: 10.1111/pbi.12538 (PMC5067613; doi:10.1111/pbi.12538)
Supplement: Supplementary file 9 — Figure S9 Quantile‐quantile plots (QQ‐plots) for GWA analysis as produced by R/GAPIT. [file PBI-14-1800-s005.pdf]

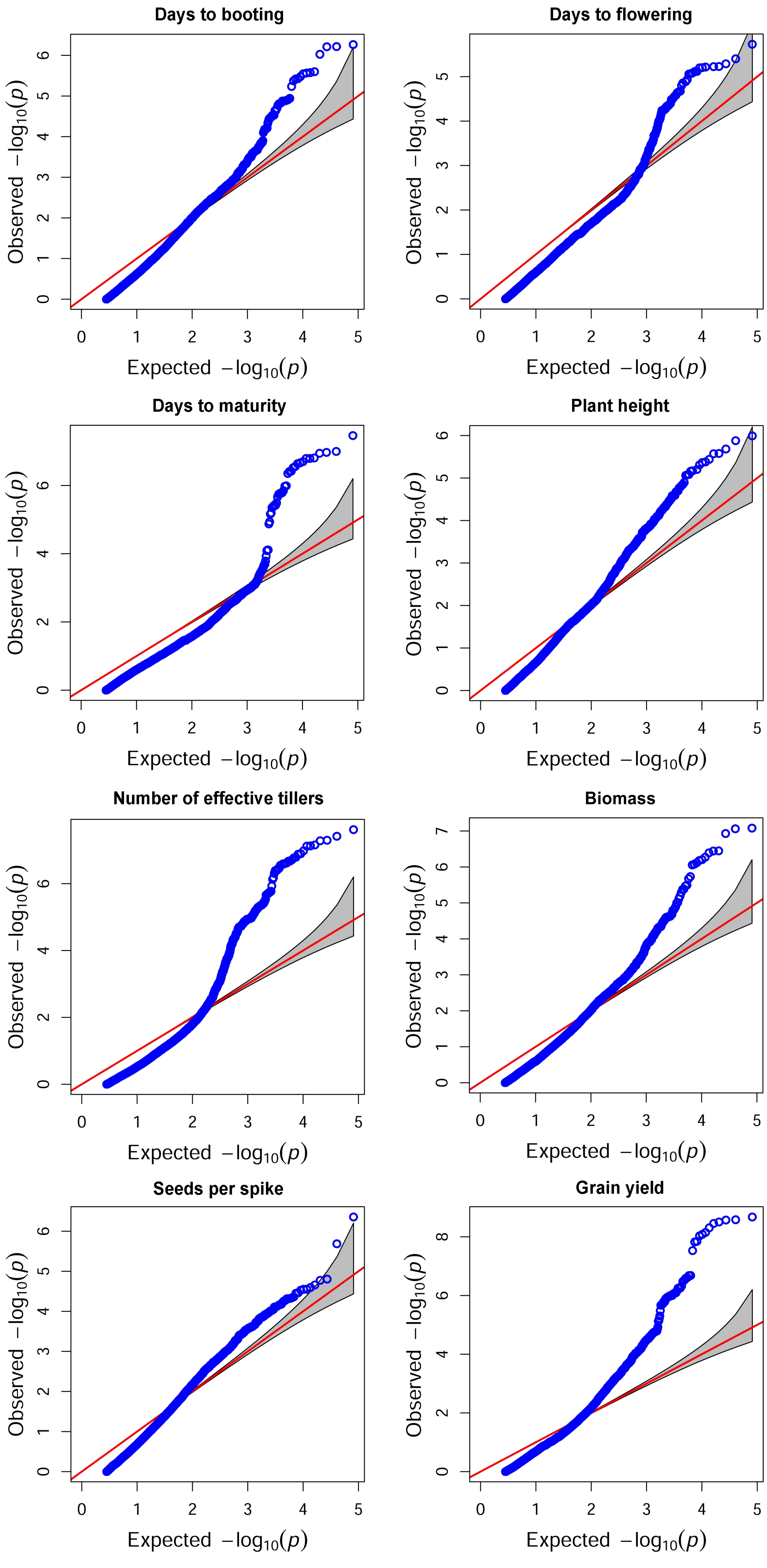

Days to booting
Days to flowering
Days to maturity
Plant height
Number of effective tillers
Biomass
Seeds per spike
Grain yield
Observed $-\log_{10}(p)$
Expected $-\log_{10}(p)$
